# Edoxaban versus warfarin in vitamin K antagonist experienced and naïve patients from the edoxaban versus warfarin in subjects undergoing cardioversion of atrial fibrillation (ENSURE-AF) randomised trial

**DOI:** 10.1007/s00392-019-01594-9

**Published:** 2020-01-08

**Authors:** Monika Kozieł, Naab Al-Saady, Søren P. Hjortshøj, Assen Goudev, Kurt Huber, Ariel Cohen, James Jin, Michael Melino, Shannon M. Winters, Andreas Goette, Gregory Y. H. Lip

**Affiliations:** 1 Liverpool Centre for Cardiovascular Science, University of Liverpool and Liverpool Heart and Chest Hospital, William Henry Duncan Building, 6 West Derby Street, Liverpool, L78TX UK; 2 Department of Cardiology, Congenital Heart Diseases and Electrotherapy, Medical University of Silesia, Silesian Centre for Heart Diseases, Zabrze, Poland; 3 Covance Inc, Cardiovascular/Metabolic, Maidenshead, UK; 4 Department of Clinical Medicine, Aalborg University, Aalborg, Denmark; 5 Queen Giovanna University Hospital, Sofia, Bulgaria; 6 3rd Medical Department, Cardiology and Intensive Care Medicine, Medcial School, Wilhelminenhospital, and Sigmund Freud University, Vienna, Austria; 7 Hôpital Saint Antoine, Hôpital Tenon; Université Pierre Et Marie Curie (Paris VI), Paris, France; 8 Daiichi Sankyo Pharma Development, Basking Ridge, New York, USA; 9 Daiichi Sankyo, Basking Ridge, New York, USA; 10 St. Vincenz-Hospital, Paderborn, Germany; 11 Working Group: Molecular Electrophysiology, University Hospital Magdeburg, Magdeburg, Germany; 12 Aalborg Thrombosis Research Unit, Department of Clinical Medicine, Aalborg University, Aalborg, Denmark

**Keywords:** Atrial fibrillation, Edoxaban, Vitamin K antagonists

## Abstract

**Background:**

In ENSURE-AF study, edoxaban had similar efficacy and safety profile versus enoxaparin–warfarin (enox–warf) in patients undergoing electrical cardioversion of non-valvular atrial fibrillation.

**Objectives:**

To evaluate the efficacy and safety of edoxaban versus enox–warf in patients who were vitamin K antagonists (VKA) naïve or experienced at time of randomisation into ENSURE-AF trial.

**Methods:**

The primary efficacy endpoint was a composite of stroke, systemic embolic event, myocardial infarction, and cardiovascular death during the overall study period, 28 days on study drug after cardioversion and 30 days follow-up. The primary safety endpoint was the composite of major and clinically relevant nonmajor bleeding during the on-medication period from time of first dose to last dose of study drug taken + 3 days.

**Results:**

Of 2199 patients enrolled in ENSURE-AF, 1095 were randomised to edoxaban and 1104 to enox–warf. There were numerically fewer primary efficacy endpoint events with edoxaban than enox–warf irrespective of whether VKA experienced or naïve (0.5% vs. 0.9%, 0.3% vs. 1.4%, respectively). There were no significant differences in the primary safety endpoint [odds ratio (OR) 2.09, 95% confidence interval (CI) 0.72–6.81 in anticoagulant experienced patients, OR 0.77, 95% CI 0.15–3.60 in anticoagulant naïve patients] and in major bleeding rates regardless of treatment or VKA experience (OR 0.69, 95%CI 0.06–6.04, OR 0.48, 95% CI 0.01–9.25, respectively).

**Conclusions:**

Edoxaban had comparable efficacy and safety to optimized anticoagulation with enox–warf. The primary efficacy and safety endpoint outcomes were broadly similar between VKA experienced or naïve patients.

## Introduction

Stroke prevention with oral anticoagulation (OAC) is one of the cornerstones of atrial fibrillation (AF) management [1]. Moreover, non-vitamin K antagonist oral anticoagulants (NOACs) are increasingly recommended as first line management for stroke prevention in AF [2, 3]. Studies assessing NOACs show that they cause less intracranial haemorrhage and life-threatening bleedings when compared to warfarin, as well as a reduction in mortality [4].

OAC significantly reduces stroke in patients undergoing electrical cardioversion (ECV) [5, 6]. Initiation of OAC is obligatory in all patients planned for cardioversion [7, 8], and permanent OAC continuation after ECV is indicated in all subjects with stroke risk factors based on CHA_2_DS_2_-VASc score (congestive heart failure, hypertension, age ≥ 75 years, diabetes, stroke/transient ischaemic attack (TIA), vascular disease, age 65–74 years, sex category) [2, 3]. In case of early ECV, transesophageal echocardiography (TEE) may be useful for excluding the majority of left atrial thrombi.

The Edoxaban versus warfarin in subjects undergoing cardioversion of atrial fibrillation (ENSURE-AF) assessed the use of edoxaban vs. enoxaparin–warfarin in patients with non-valvular AF undergoing ECV [9]. This large prospective, randomised, multicenter open-label trial showed a similar efficacy and safety profile of edoxaban vs. enoxaparin–warfarin (enox–warf) in patients with non-valvular AF. Whether this is related to prior vitamin K antagonist (VKA) exposure or not is uncertain.

The aim of this ancillary analysis from the ENSURE-AF trial was to evaluate the efficacy and safety of edoxaban vs. enox–warf in patients who were VKA naïve or experienced at the time of randomisation in the ENSURE-AF study.

## Methods

The design of the ENSURE-AF trial (NCT 02,072,434) has been previously reported [9, 10]. In brief, this study is a multicentre, prospective, randomised, open-label (patients, statisticians and investigators were not masked to treatment allocation) trial with blinded endpoint assessment. The patients with non-valvular AF (lasting from 48 h to 12 months), eligible for ECV and OAC were enrolled. The patients’ stratification was based on cardioversion approach (TEE or non-TEE), as established by the local investigator or determined by the patient’s previous experience with OAC (i.e. VKA experience vs. naive), edoxaban dose and region. Edoxaban 60 mg once daily (QD; 30 mg QD for creatinine clearance [CrCl] of 15–50 mL/min, weight ≤ 60 kg, and/or concomitant use of P-glycoprotein inhibitor) was compared with enoxaparin–warfarin in 2199 patients (randomisation 1:1; Fig. 1). Subjects with an international normalised ratio (INR) < 2.0 at randomisation were treated with enoxaparin bridging and daily warfarin until the INR was ≥ 2.0, and those with INR ≥ 2.0 at the time of randomisation did not need enoxaparin and were medicated with warfarin alone. The dosing of warfarin was adjusted to achieve and maintain the INR level from 2.0 to 3.0. INR was assessed once every 2–3 days until the value achieved the therapeutic range. Subjects in edoxaban group had to start medication at least 2 h prior ECV. The next dose of edoxaban was taken next day and then on a 24-h cycle until day 28 post cardioversion.

**Fig. 1:**
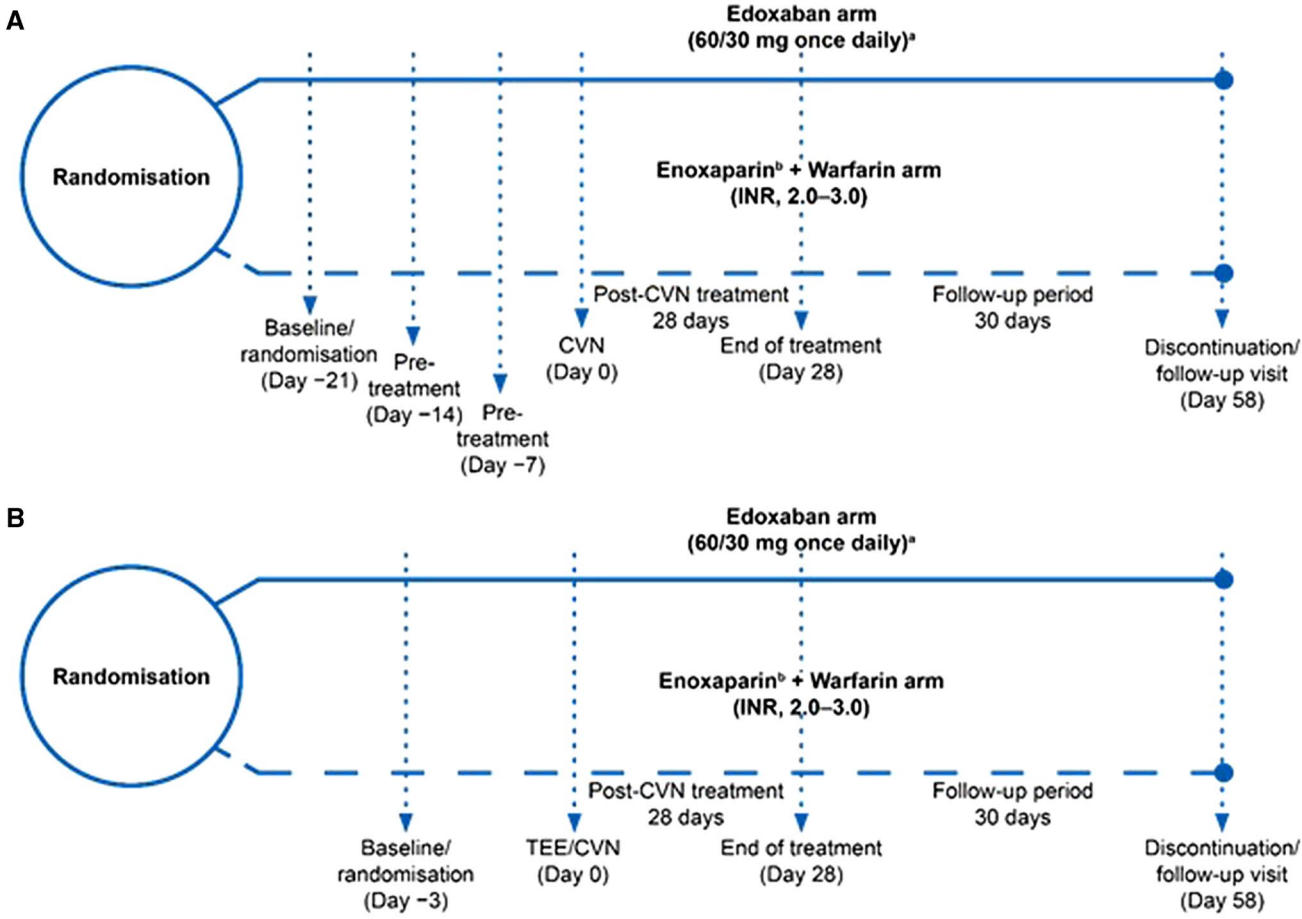
Study design for **A** non-TEE-guided stratum and **B** TEE-guided stratum in ENSURE-AF. **a** Patients meeting ≥ 1 of the following criteria were dose-reduced to 30 mg: CrCl ≥ 15 mL/min and ≤ 50 mL/min; low body weight (≤ 60 kg); or concomitant use of P-glycoprotein inhibitors. **b** Patients with INR ≥ 2 at randomisation did not require enoxaparin. *CrCl* creatinine clearance, *CVN* cardioversion, *INR* international normalised ratio, *TEE* transoesophageal echocardiography

In the TEE-guided group, TEE and ECV had to be done within 3 days of randomisation. In case of presence of thrombi on TEE, patients had a possibility of completing 28 days of study medication without ECV or being discontinued from the study.

In all patients, ECV was done at a minimum of 21 days following the start of medication. In the edoxaban group, patients were treated with edoxaban for a minimum of 21 days before cardioversion followed by the procedure and an additional 28 days of treatment. An algorithm dedicated to the treatment of patients with labile INR was included in the protocol and the 21-day pre-treatment cycle was possible to be restarted under predefined circumstances. The patients who were relocated from a previous OAC to edoxaban did so in accordance with the transition algorithm provided. The patients with spontaneous cardioversion in the preprocedural period (confirmed by a recording of sinus rhythm in electrocardiogram) were required to complete 28 days of treatment from the day that spontaneous cardioversion was recorded and 30 days of follow-up.

The study protocol is concordant with the Declaration of Helsinki and the International Conference on Harmonization consolidated guideline E6 for Good Clinical Practice (CPMP/ICH/135/95). A signed patient informed consent form was acquired before participation in the study.

The primary efficacy endpoint was defined as a composite of stroke, systemic embolic event (SEE), myocardial infarction (MI), and cardiovascular death analysed during the overall study period, 28 days on study drug after cardioversion and then follow-up was performed for safety for another 30 days after completing or discontinuing the medication. The primary safety endpoint was the composite of major and clinically relevant nonmajor bleeding (CRNM) analysed during the on-treatment period from the time of first dose to last dose of study drug taken + 3 days.

### Statistical analysis

The primary efficacy analysis was performed on the intention to treat population (all patients who were enrolled into the trial and randomly assigned). All patients who took at least one dose of study drug (the safety population) were included in primary safety analysis. Sensitivity analyses were calculated in the per-protocol population of all randomly assigned patients without any predefined major protocol deviations. Odds ratios and 95% confidence intervals are shown to evaluate the difference between treatment arms.

## Results

In this analysis, 2199 patients were enrolled from 2014 to 2015, from 239 centers in 19 countries in Europe and the United States of America: 1095 patients were randomised to edoxaban and 1104 to enox–warf. Among 1095 patients assigned to edoxaban, 1067 received allocated treatment, whilst among 1,104 patients assigned to enox–warf, 1082 received allocated treatment. From 1095 patients assigned to edoxaban, 988 (90.2%) of patients from edoxaban group and 966 (87.5%) of those from enox–warf group were cardioverted electrically or spontaneously: 600 (27.3%) of all patients were VKA naïve (not having taken any OAC within 30 days before randomisation), whilst 1599 (72.7%) of all patients were VKA experienced at randomisation. Of those undergoing cardioversion, 589 patients from the edoxaban group and 594 patients from the enox–warf group underwent TEE-guided cardioversion [9]. Baseline characteristics of patients are summarised in Table 1.

**Table 1:** Baseline demographics

	Overall		VKA experienced		VKA naïve	
	Edoxaban (*N* = 1095)	Enox–warf (*N* = 1104)	Edoxaban (*n* = 791)	Enox–warf (*n* = 808)	Edoxaban (*n* = 304)	Enox–warf (*n* = 296)
Age, years	64.3 (10.3)	64.2 (10.8)	64.6 (9.9)	64.1 (10.8)	63.5 (11.4)	64.3 (10.8)
> 65, *n* (%)	509 (46.5)	530 (48.0)	374 (47.3)	383 (47.4)	135 (44.4)	147 (49.7)
Weight, kg	90.9 (18.3)	91.2 (19.0)	90.6 (18.4)	90.9 (19.0)	91.5 (18.1)	91.8 (19.0)
≤ 60, *n* (%)	21 (1.9)	33 (3.0)	17 (2.2)	21 (2.6)	4 (1.3)	12 (4.1)
Anticoagulant experienced, *n* (%)						
Current VKA user^a^	513 (46.8)	558 (50.5)	484 (61.2)	529 (65.5)	29 (9.5)	29 (9.8)
Current NOAC user^a^	157 (14.3)	148 (13.4)	145 (18.3)	133 (16.5)	12 (3.9)	15 (5.1)
CrCl	94.0 (35.7)	94.1 (34.7)	92.5 (34.9)	92.9 (34.6)	97.8 (37.8)	97.5 (34.8)
TtTR (days)	–	7.7 (5.1)	–	7.4 (5.4)	–	8.1 (4.5)
TiTR (% of time)	–	70.8 (27.4)	–	72.0 (26.8)	–	67.4 (28.9)
TTR (% of time)^b^	–	59.8 (30.6)	–	61.8 (30.6)	–	54.4 (30.1)
Medical history						
Congestive HF	476 (43.5)	484 (43.8)	365 (46.1)	376 (46.5)	111 (36.5)	108 (36.5)
CAD	181 (16.5)	197 (17.8)	133 (16.8)	149 (18.4)	48 (15.8)	48 (16.2)
Hypertension	850 (77.6)	864 (78.3)	637 (80.5)	633 (78.3)	213 (70.1)	231 (78.0)
Diabetes	218 (19.9)	197 (17.8)	159 (20.1)	136 (16.8)	59 (19.4)	61 (20.6)
PAD	40 (3.7)	54 (4.9)	29 (3.7)	43 (5.3)	11 (3.6)	11 (3.7)
VHD	250 (22.8)	240 (21.7)	194 (24.5)	179 (22.2)	56 (18.4)	61 (20.6)
ICH	2 (0.2)	3 (0.3)	1 (0.1)	3 (0.4)	1 (0.3)	0
Ischaemic stroke/TIA	68 (6.2)	66 (6.0)	54 (6.8)	50 (6.2)	14 (4.6)	16 (5.4)
MI	69 (6.3)	78 (7.1)	51 (6.4)	56 (6.9)	18 (5.9)	22 (7.4)
Life-threatening bleed	3 (0.3)	3 (0.3)	2 (0.3)	2 (0.2)	1 (0.3)	1 (0.3)
AF history, *n* (%)						
Paroxysmal (≤ 7 days)	208 (19.0)	207 (18.8)	103 (13.0)	119 (14.8)	105 (34.5)	88 (29.8)
Persistent (> 7 days, < 1 yr)	887 (81.0)	890 (80.6)	688 (87.0)	683 (85.2)	199 (65.5)	207 (70.2)
CHA_2_DS_2_-VASc score	2.6 (1.49)	2.6 (1.40)	2.7 (1.5)	2.6 (1.4)	2.4 (1.5)	2.6 (1.4)
HAS-BLED score	0.9 (0.78)	0.9 (0.79)	0.9 (0.8)	0.9 (0.8)	0.8 (0.8)	0.8 (0.8)
Prior drug therapies						
Aspirin	192 (17.4)	221 (20.0)	105 (13.3)	130 (16.1)	87 (28.6)	91 (30.7)
Statins	429 (39.2)	411 (37.2)	320 (40.5)	302 (37.4)	109 (35.9)	109 (36.8)
ACEI/ARB	692 (63.2)	688 (62.3)	525 (66.4)	513 (63.5)	167 (54.9)	175 (59.1)
Beta blocker	862 (78.7)	847 (76.7)	640 (80.9)	622 (77.0)	222 (73.0)	225 (76.0)

Data provided as mean (standard deviation) unless otherwise indicated

*ACEI/ARB* angiotensin converting enzyme inhibitor/angiotensin receptor blocker, *AF* atrial fibrillation, *CAD* coronary artery disease, *CHA*_*2*_*DS*_*2*_*-VASc* congestive heart failure, hypertension, age ≥ 75, diabetes mellitus, and prior stroke or transient ischaemic attack or thromboembolism, vascular disease, age 65–74 years, sex category; *CrCl* creatinine clearance, *Enox–warf* enoxaparin–warfarin, *HAS-BLED* hypertension, abnormal renal and liver function, stroke, bleeding history or disposition, labile INR, elderly, drugs or alcohol, *HF* heart failure, *ICH* intracranial haemorrhage, *MI* myocardial infarction, *NOAC* novel oral anticoagulant, *PAD* peripheral artery disease, *TIA* transient ischaemic attack, *TiTR* time in therapeutic range (calculated from the first day with 2 ≤ INR ≤ 3), *TTR* time in therapeutic range (calculated from day 8 of study drug), *TtTR* time to therapeutic range, *VHD* valvular heart disease, *VKA* vitamin K antagonist

^a^Current defined as using VKA or NOAC at randomisation or within 30 days prior to randomisation

^b^Rosendaal method

1 patient of 2199 (< 1%) was lost to follow-up.

The primary efficacy endpoint appeared in 11 (0.7%) of the VKA experienced group and in 5 (0.8%) of the VKA naïve group [odds ratio (OR) 0.58, 95% confidence interval (CI) 0.12–2.30 in VKA experienced patients, OR 0.24, 95% CI 0.0–2.46 in VKA naïve patients), Table 2. The composite of major and CRNM bleeding events occurred in 18 (1.2%) of VKA experienced patients and in 9 (1.5%) of VKA naïve patients (OR 2.09, 95% CI 0.72–6.81 in VKA experienced patients, OR 0.77, 95% CI 0.15–3.60 in VKA naïve patients), Table 2.

**Table 2:** Event rates according to prior VKA experience classification and treatment

	Overall		VKA experienced		VKA naïve	
	Edoxaban	Enox-warf	Edoxaban	Enox-warf	Edoxaban	Enox-warf
First stroke, SEE, MI, or CV mortality^a^						
* N*	1095	1104	791	808	304	296
*n* (%)	5 (0.5)	11 (1.0)	4 (0.5)	7 (0.9)	1 (0.3)	4 (1.4)
OR (95% CI)	0.46 (0.12, 1.43)		0.58 (0.12, 2.30)		0.24 (0.00, 2.46)	
Major or CRNM bleeding events^b^						
* N*	1067	1082	764	791	303	291
*n* (%)	16 (1.5)	11 (1.0)	12 (1.6)	6 (0.8)	4 (1.3)	5 (1.7)
OR (95% CI)	1.48 (0.64, 3.55)		2.09 (0.72, 6.81)		0.77 (0.15, 3.60)	
Major bleeding events^b^						
* N*	1067	1082	764	791	303	291
*n* (%)	3 (0.3)	5 (0.5)	2 (0.3)	3 (0.4)	1 (0.3)	2 (0.7)
OR (95% CI)	0.61 (0.09, 3.13)		0.69 (0.06, 6.04)		0.48 (0.01, 9.25)	

*CI* confidence interval, *CRNM* clinically relevant nonmajor, *CV* cardiovascular, *Enox–warf* enoxaparin–warfarin, *MI* myocardial infarction, *OR* odds ratio, *SEE* systemic embolic event, *VKA* vitamin K antagonist

^a^Intent to treat population, overall study period

^b^All treated patients, on-treatment period

Major bleeding appeared in five (0.3%) of VKA experienced patients and in three (0.5%) of VKA naïve patients (OR 0.69, 95% CI 0.06–6.04 in VKA experienced patients, OR 0.48, 95% CI 0.01–9.25 in VKA naïve patients), Table 2.

## Discussion

The ENSURE-AF provides the largest prospective trial dataset for edoxaban in terms of non-valvular AF patients scheduled for cardioversion. In this ancillary analysis from ENSURE-AF, our findings are as follows: (1) edoxaban had non-significantly different rates of primary efficacy and safety endpoint outcomes compared with enoxaparin–warfarin, (2) VKA experienced patients had non-significantly different primary efficacy and safety endpoint outcomes to VKA naïve subjects.

Peri-cardioversion anticoagulation with VKA is associated with lower risk of stroke or thromboembolism than no anticoagulation [11]. Unfortunately, major bleeding events were not evaluated in the above-mentioned systematic review of observational studies.

Planned ECV at study entry was an exclusion criterion in phase 3 randomised trials which compared NOAC to warfarin; however, ECV was performed in those trials during their course [12]. In a retrospective analysis of data from the randomised evaluation of long-term anticoagulation therapy (RE-LY), the proportion of stroke and major bleeding within 30 days of cardioversion on dabigatran (150 mg twice daily and 110 mg twice daily) were low and similar to those on warfarin with or without TEE guidance [12, 13]. In other retrospective analysis [14], there were no differences in the incidence of stroke or systemic embolism and death in patients medicated with rivaroxaban and warfarin. Patients after cardioversion in the apixaban for reduction in stroke and other thromboembolic events in atrial fibrillation (ARISTOTLE) [15] were also evaluated retrospectively. The prevalence of MI, major bleeding and death was similar in patients treated with apixaban and warfarin. In the effective anticoagulation with factor Xa next generation (ENGAGE) AF-TIMI 48 trial [16], stroke or SEE, major bleeding or death were infrequent and similar in patients medicated with edoxaban and warfarin [17]. However, these studies are limited by its post-hoc, nonrandomized design, small cohort and lack of TEE data.

Explore the efficacy and safety of once-daily oral rivaroxaban for the prevention of cardiovascular events in patients with non-valvular atrial fibrillation scheduled for cardioversion (X-VeRT) [18] was a randomised, open-label prospective study of rivaroxaban in patients with AF undergoing elective cardioversion. Rivaroxaban (20 mg QD or 15 mg QD in patients with moderate renal impairment) was associated with low thromboembolic and bleeding risks and broadly similar to risks in patients treated with VKA. Of note, in the group of patients, where cardioversion was performed within the target time range of 21–25 days after randomisation, only 36.3% of patients on warfarin were cardioverted within the target time due to inadequate coagulation.

Another prospective trial, E**l**iquis evaluated in acute cardioversion compared to usual treatments for anticoagulation in subjccts with atrial fibrillation (EMANATE) [19], showed low rates of stroke, systemic embolic events, death and bleeding events in both the apixaban and heparin/VKA-treated patients. In this study, patients with AF were scheduled for cardioversion during anticoagulation with either apixaban or a conventional heparin/VKA regimen and anticoagulation lasted ≤ 48 h prior to randomisation. The main limitation of the study is the descriptive design, without hypothesis testing, and power calculations.

In our study, the primary efficacy endpoint outcomes were similar in VKA experienced and naïve patients.

### Strengths and limitations

Our study has some limitations that should be noted. This trial was underpowered to present statistically significant differences for efficacy or safety endpoints. Moreover, the open-label study design may be associated with bias in reporting outcome. Strengths of the study are the prospective trial design which is the largest dataset comparing a NOAC (edoxaban) to warfarin for efficacy and safety in the peri-cardioversion period. Enox–warf therapy was optimally used with acceptable time in therapeutic range. Management with edoxaban was associated with compliance of more than 99%.

## Conclusions

Edoxaban had comparable efficacy and safety to optimized anticoagulation with enox–warf. The primary efficacy and safety endpoint outcomes were broadly similar between VKA experienced or naïve patients.

## References

[1] Lip G, Freedman B, De Caterina R, Potpara TS (2017). Stroke prevention in atrial fibrillation: Past, present and future. Comparing the guidelines and practical decision-making. Thromb Haemost.

[2] Kirchhof P, Benussi S, Kotecha D, Ahlsson A, Atar D, Casadei B (2016). 2016 ESC guidelines for the management of atrial fibrillation developed in collaboration with EACTS. Eur Heart J.

[3] Lip GYH, Banerjee A, Boriani G, Chiang CE, Fargo R, Freedman B (2018). Antithrombotic therapy for atrial fibrillation: CHEST guideline and expert panel report. Chest.

[4] Ruff CT, Giugliano RP, Braunwald E, Hoffman EB, Deenadayalu N, Ezekowitz MD (2014). Comparison of the efficacy and safety of new oral anticoagulants with warfarin in patients with atrial fibrillation: a meta-analysis of randomised trials. Lancet.

[5] Airaksinen KE, Gronberg T, Nuotio I, Nikkinen M, Ylitalo A, Biancari F (2013). Thromboembolic complications after cardioversion of acute atrial fibrillation: the FinCV (Finnish CardioVersion) study. J Am Coll Cardiol.

[6] Hansen ML, Jepsen RM, Olesen JB, Ruwald MH, Karasoy D, Gislason GH (2015). Thromboembolic risk in 16 274 atrial fibrillation patients undergoing direct current cardioversion with and without oral anticoagulant therapy. Europace.

[7] Schmidt-Lucke C, Paar WD, Stellbrink C, Nixdorff U, Hofmann T, Meurer J (2007). Quality of anticoagulation with unfractionated heparin plus phenprocoumon for the prevention of thromboembolic complications in cardioversion for non-valvular atrial fibrillation. Sub-analysis from the Anticoagulation in Cardioversion using Enoxaparin (ACE) trial. Thromb Res.

[8] Schadlich PK, Schmidt-Lucke C, Huppertz E, Lehmacher W, Nixdorff U, Stellbrink C (2007). Economic evaluation of enoxaparin for anticoagulation in early cardioversion of persisting nonvalvular atrial fibrillation: a statutory health insurance perspective from Germany. Am J Cardiovasc Drugs.

[9] Goette A, Merino JL, Ezekowitz MD, Zamoryakhin D, Melino M, Jin J (2016). Edoxaban versus enoxaparin-warfarin in patients undergoing cardioversion of atrial fibrillation (ENSURE-AF): a randomised, open-label, phase 3b trial. Lancet.

[10] Lip GY, Merino J, Ezekowitz M, Ellenbogen K, Zamoryakhin D, Lanz H (2015). A prospective evaluation of edoxaban compared to warfarin in subjects undergoing cardioversion of atrial fibrillation: the Edoxaban vs. warfarin in subjects undergoing cardioversion of atrial fibrillation (ENSURE-AF) study. Am Heart J.

[11] Moreyra E, Finkelhor RS, Cebul RD (1995). Limitations of transesophageal echocardiography in the risk assessment of patients before nonanticoagulated cardioversion from atrial fibrillation and flutter: an analysis of pooled trials. Am Heart J.

[12] Nagarakanti R, Ezekowitz MD, Oldgren J, Yang S, Chernick M, Aikens TH (2011). Dabigatran versus warfarin in patients with atrial fibrillation: an analysis of patients undergoing cardioversion. Circulation.

[13] Connolly SJ, Ezekowitz MD, Yusuf S, Eikelboom J, Oldgren J, Parekh A (2009). Dabigatran versus warfarin in patients with atrial fibrillation. N Engl J Med.

[14] Piccini JP, Stevens SR, Lokhnygina Y, Patel MR, Halperin JL, Singer DE (2013). Outcomes after cardioversion and atrial fibrillation ablation in patients treated with rivaroxaban and warfarin in the ROCKET AF trial. J Am Coll Cardiol.

[15] Flaker G, Lopes RD, Al-Khatib SM, Hermosillo AG, Hohnloser SH, Tinga B (2014). Efficacy and safety of apixaban in patients after cardioversion for atrial fibrillation: insights from the ARISTOTLE trial (apixaban for reduction in stroke and other thromboembolic events in atrial fibrillation). J Am Coll Cardiol.

[16] Plitt A, Ezekowitz MD, De Caterina R, Nordio F, Peterson N, Giugliano RP (2016). Cardioversion of atrial fibrillation in ENGAGE AF-TIMI 48. Clin Cardiol.

[17] Giugliano RP, Ruff CT, Braunwald E, Murphy SA, Wiviott SD, Halperin JL (2013). Edoxaban versus warfarin in patients with atrial fibrillation. N Engl J Med.

[18] Cappato R, Ezekowitz MD, Klein AL, Camm AJ, Ma CS, Le Heuzey JY (2014). Rivaroxaban vs. vitamin K antagonists for cardioversion in atrial fibrillation. Eur Heart J.

[19] Ezekowitz MD, Pollack CV, Halperin JL, England RD, VanPelt NS, Spahr J (2018). Apixaban compared to heparin/vitamin K antagonist in patients with atrial fibrillation scheduled for cardioversion: the EMANATE trial. Eur Heart J.

